# Biomimetic vs. Direct Approach to Deposit Hydroxyapatite on the Surface of Low Melting Point Polymers for Tissue Engineering

**DOI:** 10.3390/nano10112162

**Published:** 2020-10-29

**Authors:** Andri K. Riau, Subbu S. Venkatraman, Jodhbir S. Mehta

**Affiliations:** 1 Tissue Engineering and Cell Therapy, Singapore Eye Research Institute, Singapore 169856, Singapore; 2 School of Materials Science and Engineering, Nanyang Technological University, Singapore 639798, Singapore; 3 Ophthalmology and Visual Sciences Academic Clinical Program, Duke-NUS Medical School, Singapore 169857, Singapore; 4 Department of Materials Science and Engineering, National University of Singapore, Singapore 119077, Singapore; subbu@nus.edu.sg; 5 Singapore National Eye Centre, Singapore 169856, Singapore

**Keywords:** hydroxyapatite, polymer, simulated body fluid, dip-coating, tissue engineering, crystallinity

## Abstract

Polymers are widely used in many applications in the field of biomedical engineering. Among eclectic selections of polymers, those with low melting temperature (T_m_ < 200 °C), such as poly(methyl methacrylate), poly(lactic-co-glycolic acid), or polyethylene, are often used in bone, dental, maxillofacial, and corneal tissue engineering as substrates or scaffolds. These polymers, however, are bioinert, have a lack of reactive surface functional groups, and have poor wettability, affecting their ability to promote cellular functions and biointegration with the surrounding tissue. Improving the biointegration can be achieved by depositing hydroxyapatite (HAp) on the polymeric substrates. Conventional thermal spray and vapor phase coating, including the Food and Drug Administration (FDA)-approved plasma spray technique, is not suitable for application on the low T_m_ polymers due to the high processing temperature, reaching more than 1000 °C. Two non-thermal HAp coating approaches have been described in the literature, namely, the biomimetic deposition and direct nanoparticle immobilization techniques. In the current review, we elaborate on the unique features of each technique, followed by discussing the advantages and disadvantages of each technique to help readers decide on which method is more suitable for their intended applications. Finally, the future perspectives of the non-thermal HAp coating are given in the conclusion.

## 1. Introduction

The uses of polymers are ubiquitous in today’s world due to their low synthesis cost, tunable mechanical properties to suit the intended application, non-toxic degradation products, and ease of manufacturing [1,2,3,4,5,6]. One of the most widespread uses of polymers can be found in the field of biomedical engineering as implants or tissue engineering products [1,2,3,4,5,6]. In many fields, polymeric materials satisfy the requirements of many biomedical applications. However, many polymers have a surface that is bioinert and deficient of free reactive functional groups (e.g., –COOH and –NH_2_), lacks topographical features, and has poor wettability, affecting their ability to promote cellular functions and biointegration with the surrounding tissue [7,8,9,10]. These surface properties often render polymers less suitable than natural biomaterials, e.g., collagen or gelatin, as implants for tissue regeneration. The clinical successes of most of such implants heavily depend on sustained material-cell interactions or bioactivity to facilitate the integration process with the surrounding host tissue [11,12]. Poor biointegration could lead to device failure or extrusion, which often requires repeat surgeries to replace the loose implants. Surface engineering to create nanoscale or microscale layers of controlled chemical composition, topography and roughness, and balanced hydrophilicity/hydrophobicity on polymeric implants have emerged as a simple, useful, and versatile approach to alleviate the aforementioned biointegration issue [9,13,14]. Another appeal of surface engineering is that the material’s property improvements can be achieved without significant alteration of the bulk properties of the implantable devices.

One of the most widely used applications in surface engineering has been the use of hydroxyapatite (HAp) coating on orthopedic, dental, and middle ear implant surfaces [15,16,17,18]. HAp is a type of calcium phosphate (CaP) bioceramics and has attracted the most attention due to its close resemblance to the chemical and mineral components of teeth and bone. It has also been described as a bioactive material due to its inherent ability to induce specific biological reactions from cells or living tissues [19,20,21]. As a result of this similarity, HAp has shown good biocompatibility with bone and tooth, and somewhat surprisingly, with the cornea [16,20,22]. In bone tissue engineering, HAp coating has been shown to enhance bone apposition to orthopedic implants, where it prevents the formation of loose fibrous tissue, but instead forms an extremely thin, epitaxial bonding layer with the bone [23,24]. Although HAp coating has not been applied to commercially available corneal prostheses, studies have shown that the bioactive material can enhance biocompatibility, adhesion, and proliferation of corneal stromal fibroblasts in vitro [16,22,25]. The HAp has also been demonstrated to be safe when implanted in vivo [16,26].

HAp coating is regularly applied to metals in load-bearing devices [27]. For this purpose, various methods have been used to deposit HAp coatings, such as thermal spraying, which includes plasma spray [28], flame spray [29], and high-velocity oxygen fuel (HVOF) spray techniques [30], sputter coating [31], electron beam deposition [32], electrophoretic deposition [33], hot isostatic pressing [34], and sol-gel methods [35]. Among them, the plasma spray has been the most widely applied coating technique in dentistry and orthopedics [36]. It is also currently the only U.S. Food and Drug Administration (FDA)-approved method for applying HAp coating on metallic implant surfaces. A common feature of the abovementioned techniques is high processing and/or annealing temperature that can reach a temperature above 1000 °C. This obviously limits their application for biomaterials with relatively low melting temperature (T_m_), such as poly(methyl methacrylate) (PMMA; T_m_ = 160 °C), poly(ethylene glycol) (PEG; T_m_ = 60 °C), polylactic acid (PLA; T_m_ = 160 °C), and poly(ε-caprolactone) (PCL; T_m_ = 60 °C), to name a few [37]. In addition, methods, such as thermal spraying and sputter coating, can only be applied on surfaces that are in the line of sight and, therefore, are not amenable for coating devices with complex dimensions or with pores [38]. Some of these techniques also require expensive and elaborate equipment to perform.

Although the clinical application of low T_m_ polymers in load-bearing prostheses is uncommon, other applications, including as scaffolds for bone, middle ear, and dental tissue regeneration and craniofacial reconstruction, are regularly studied [6,39,40,41]. Hence, a non-thermal method to deposit HAp on these polymeric substrates is of interest. A facile, non-thermal approach to deposit HAp is also particularly appealing for application on corneal prostheses, which are typically constructed with an acrylic optic cylinder (e.g., PMMA) that acts as the substitute window to the eye [9,16,22]. In the current review, we discuss two different non-thermal HAp coating approaches, namely, the biomimetic deposition and direct nanoparticle immobilization approaches, for low T_m_ polymeric substrates. We also discuss the advantages and limitations of each approach to help readers decide on which particular method is more suitable for their intended applications. We end the review with a summary and future perspective of non-thermal HAp coating.

## 2. General Considerations for Review

A keyword “calcium + phosphate + deposits” search on PubMed, conducted on 24 October 2020 yielded 2043 publications. The majority of these publications studied CaP deposits on the surface of high T_m_ materials, such as metals or bioceramics. Narrowing down the search with a keyword of “calcium + phosphate + polymer + deposits” resulted in 404 publications. Among these publications, we selected research articles that studied CaP deposits on polymers scaffolds or thin films with a T_m_ of <200 °C as the focus of the current review article. The rationale to focus on this group of biomaterials was that a large number of tissue engineering scaffolds or films are made of polymers with such intrinsic thermal property.

## 3. Limitations of Thermal Spray and Other Conventional Hydroxyapatite Coating Techniques

### 3.1. Thermal Spray

The basic mechanism of thermal spray warrants a brief discussion to understand the incompatibility of the method in depositing HAp on low T_m_ polymers. Before the application of the coating, the surface of the substrate is cleaned and abraded to facilitate the adhesion of the oncoming HAp particles. Several surface cleaning methods have been described: chemical etching, mechanical preparation, electrical cleaning, laser etching, and grit blasting using abrasive materials, including silicon carbide or corundrum [42,43,44].

The coating deposition involves the projection of melted HAp powder that is injected in the flame or plasma stream onto the surface of the substrate. The coating process, hence, relies on two energy sources: thermal energy to melt (or partially melt) the HAp powder and kinetic energy to project and accelerate the HAp onto the substrate [45,46]. In plasma spray, the plasma temperature reaches above 10,000 °C and the coating materials are projected at a velocity of about 150–600 m/s, while in HVOF, the flame temperature reaches approximately 3000 °C, albeit a higher particle projection velocity (400–1000 m/s) is required to produce a similar coating outcome to the former technique [47]. The kinetic energy supplies deformation energy for the HAp particles after the impact with the substrate (the particles assume a “splat” shape to fill the surface irregularities that are prepared before the coating deposition) and generates heat due to non-elastic impacts [48]. The coating forms through the overlapping of multiple layers of the coating material. The coating adhesion is generally mechanical on metallic surfaces, apart from some areas where a local melting and diffusion can occur with the substrate [48].

### 3.2. Vapor Deposition

The vapor deposition technique is generally categorized into either physical or chemical vapor deposition. Physical vapor deposition (PVD) is the process of forming a thin HAp coating, consisting of submicron particles, on a substrate as the result of evaporation of a HAp target into calcium and phosphate ions by plasma, arc discharge, or mechanical removal from the target [49]. To facilitate the process of evaporation, PVD usually takes place in a vacuum chamber. Depending on the technique used to knock the ions off the target, PVD methods are identified as pulsed electron deposition if the ions are pulled out from the target through collisions with electrons [50], or as pulsed laser deposition if the method uses a high-power laser beam to bombard the target, resulting in a gaseous phase that consists of atoms and ions, which propel towards the substrate as a plasma plume [51]. Another frequently studied PVD method to deposit HAp is magnetron sputtering, which involves the ejection of calcium and phosphate ions from the HAp target by powerful magnets, which are then propelled towards the substrate [52]. Following these methods, a high annealing temperature is needed to improve the crystallization of the HAp coating.

In contrast to PVD, chemical vapor deposition (CVD) is more suitable for the deposition of coating materials on substrates with complex geometry. CVD utilizes chemical reactions of a precursor gas in a heated chamber containing the substrate [53]. The products of the chemical reactions are deposited in thin layers on the surface of the substrate. Following this, the volatile by-products are exhausted from the system. An economic-related drawback of both the PVD and CVD is that they require highly controlled equipment and vacuum chambers. Moreover, a specialized facility to handle the high-temperature deposition process and the volatile gases in CVD is required to house the equipment, which further increases the process costs.

### 3.3. Hot Isostatic Pressing

Hot isostatic pressing is a process that subjects a substrate (to be coated with HAp) to elevated temperature from several hundreds to 2000 °C and gas pressure from several tens to 200 MPa in a high-pressure containment vessel. To perform deposition, initially, substrates are covered by HAp powder. Both organic binders and some other additives are usually used to improve fixation. The specimens are then heated and simultaneously pressed, forcing the powder to integrate into the substrate [54]. However, the majority of the HAp deposits produced by the technique are often contaminated by metals and SiO_2_ particles, due to the use of glass encapsulating tubes [55]. Furthermore, it is difficult to coat complex substrates by this method.

### 3.4. Sol-Gel Deposition and Dip-Coating

Sol-gel deposition does not require extremely high coating deposition temperatures but tends to produce amorphous and non-stoichiometric HAp. Calcination or annealing of the coating at a temperature of at least 600 °C is, therefore, necessary to enhance the crystallinity, as well as to remove moisture and residual solvents, ammonia, or carbonates from the preparatory steps [56,57]. Due to the fluidity of the “sol” component, sol-gel deposition can be employed to achieve uniform HAp coating throughout a porous substrate and substrate with complex geometry [58]. The sol-gel method involves dipping substrates into the solution, containing supersaturated calcium and phosphate, and allowing the coating to dry to form a viscous gel-like layer [59]. The gel-like coating can be annealed to form a hardened layer of HAp with high crystallinity on the substrate.

An extension of the sol-gel deposition method is the dip-coating method, which involves immersing of the substrate into a solution containing HAp precursors that are soluble salts of the cations (e.g., Ca(NO_3_)_2_.4H_2_O) and alkoxides of the anions (e.g., P(OCH_2_CH_3_)_3_ and Si(OCH_2_CH_3_)_4_) [60]. The substrate is typically dipped at a constant speed and the coating is deposited during the substrate withdrawal. After drying, solid HAp deposits become adhered to the substrate. The pulling up speed determines the thickness of the coating: the faster the withdrawal, the thinner the coating. To increase the thickness, the dip-and-dry cycle can be multiplied [61].

### 3.5. Electrophoretic Deposition

Another technique that does not require HAp deposition in high temperatures is solution-based electrophoretic deposition [33]. An electrophoretic deposition involves the migration of charged particles toward the implant, to which an opposite charge has been applied. The deposition occurs via the coagulation of particles and polymers into a dense composite (e.g., HAp and chitosan) film on the charged substrate [62,63]. The nature of the technique, however, requires an electrically conductive material, such as a metallic substrate to work. The resulting CaP coating is typically amorphous and still requires calcination at high temperatures to improve its crystallinity.

## 4. Non-Thermal Hydroxyapatite Coating Methods

It is obvious that the thermal spray and vapor deposition techniques do not apply to most polymers, let alone low T_m_ polymers. First, the abrasion methods in the surface preparation stage are typically harsh, intended to increase the surface area and create microscale roughness for the coating to mechanically adhere. As such, these treatments may not be well tolerated by low T_m_ or soft polymers. Second and the confounding factor of the techniques is the extremely high processing temperature. The high particle projection velocity may also not be able to be tolerated by the soft polymers. Other techniques that are solution-based and non-thermal at the deposition phase eventually require a calcination step to densify and crystallize the HAp coating.

### 4.1. Biomimetic Approach

The biomimetic HAp coating process overcomes many of the shortcomings of conventional thermal spray coating techniques and mimics nature’s biomineralization mechanism [64]. In nature, organisms use proteins and organic materials (polymers) as templates for the formation of mineral structures, such as teeth, bones, and shells. The combination of protein and polymers control the mineralization rate, mineral phase, and orientation of HAp crystals. In humans, biomineralization typically occurs at physiological temperature (~37 °C) and neutral pH range. Researchers have extrapolated this natural mineralization method and subsequently developed a solution-based process that mimics nature’s template-mediated materialization. Such an approach can be applied to any surface that interfaces with an aqueous solution. This benign, non-thermal coating process can, therefore, be easily applied to implants with pores and complex dimensions.

In 1990, Kokubo et al. demonstrated that the formation of an apatite layer on bioactive ceramics can be reproduced by incubating a substrate in simulated body fluid (SBF) in vitro [65]. SBF is a solution that has inorganic ion concentrations similar to those of human blood plasma but does not contain any cells or protein. The solution contains supersaturated levels of calcium (Ca^2+^) and hydrogen phosphate (HPO_4_^2−^) ions. The pH of SBF is typically adjusted to 7.25–7.40 at 36.5 °C. There have been several versions of SBF, differing in the concentrations of the components and buffer solutions [65,66,67,68,69,70]. Over the years, to accelerate the mineralization process, some researchers have increased the ion concentrations of SBF to up to 10 times of the blood plasma (Table 1) [71,72,73]. Nevertheless, the majority of studies in the literature appeared to favor the uses of c-SBF (142.0 mM Na^+^, 5.0 mM K^+^, 1.5 mM Mg^2+^, 2.5 mM Ca^2+^, 147.8 mM Cl^−^, 4.2 mM HCO^3−^, 1.0 mM HPO_4_^2−^, and 0.5 mM SO_4_^2−^) and 1.5× SBF solutions.

**Figure 1:**
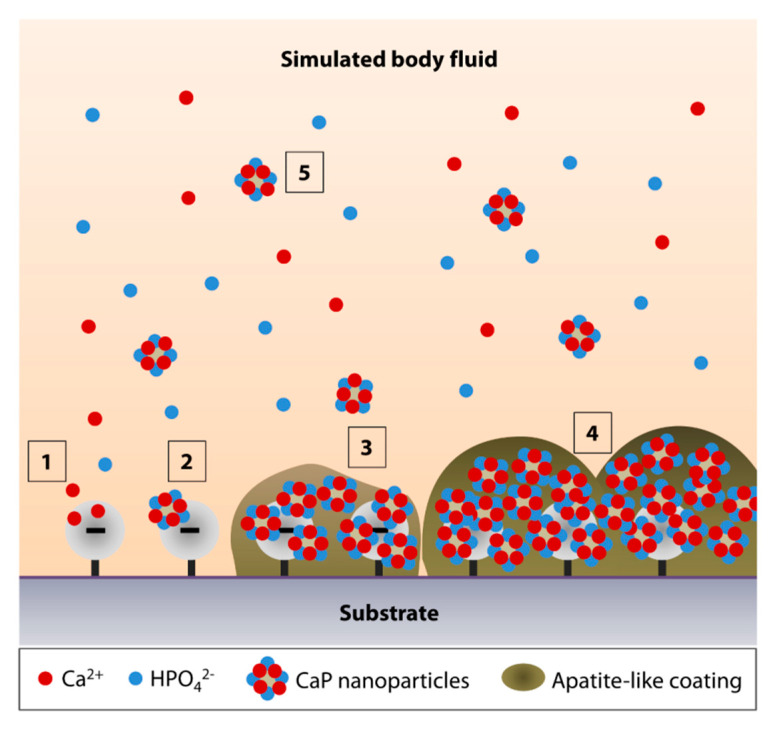
Simulated body fluid (SBF)-mediated mineralization on a negatively charged substrate surface. (**1**) Ionic interactions of calcium ions (Ca^2+^) with a negatively charged surface initiate the apatite nucleation. (**2**) Accumulation of Ca^2+^ attracts the hydrogen phosphate (HPO_4_^2−^) ions. (**3**,**4**) The accumulation forms CaP nanoparticles or crystals, serving as a secondary nucleation site for continued apatite growth. (**5**) The surface nucleation site may also attract the CaP crystals that undergo homogenous nucleation in the SBF.

The mechanism of apatite formation in SBF is fundamentally simple and is best explained by Tanahashi and Matsuda’s work that demonstrated that the apatite nucleation on a substrate in SBF is initiated by adsorption of Ca^2+^ on negatively charged surfaces, followed by the recruitment of HPO_4_^2−^ via ionic interactions with Ca^2+^, to form CaP crystals or nanoparticles (Figure 1, steps 1 and 2) [74]. Over time, the accumulation of the nanoparticles forms an apatite-like layer on the substrate (Figure 1, steps 3 and 4). The authors showed that, in decreasing order, the efficiency of apatite formation is achieved by the functionalization of substrate surfaces with –H_2_PO_4_ > –COOH > –OH > –NH_2_ > –CH_3_. In addition to heterogeneous nucleation on material surfaces, homogenous apatite nucleation can happen spontaneously in the SBF (Figure 1, step 5) [67]. Hence, the authors did not rule out the possibility of ionic interactions of the Ca^2+^ or PO_4_^3−^ of the CaP nanoparticles with the growing apatite deposits on the substrate [74].

In light of the earlier study by Tanahashi and Matsuda [74], it is apparent that surface functionalization is required to prime the surface of most biomedical polymers to increase the efficiency of CaP deposition. A later study by Leonor et al. revealed that in addition to surface phosphorylation and carboxylation, surface functionalization with sulfonic acid (–SO_3_H) is an alternative method to enhance apatite formation on low T_m_ polymers, e.g., ethylene vinyl alcohol (EVOH) and high molecular weight polyethylene (HMWPE) [75]. The sulfonated polyamide surface appeared to be more effective in driving the biomineralization process compared to the carboxylated surface [76].

#### 4.1.1. Biomineralization on Phosphorylated Surface

The most common technique to functionalize the surface of polymers with phosphonate groups is by the grafting of mono(2-acryloyloxyethyl) phosphate (MAEP) or 2-(methacryloyloxy) ethyl phosphate (MOEP) [77,78,79,80,81,82]. By grafting MOEP on high-density polyethylene (HDPE), Tretinnikov and colleagues showed the formation of an apatite-like coating could be seen as early as 2 days following incubation in c-SBF at 37 °C [78]. They also revealed that to produce a coating that was close to the theoretical Ca/P ratio of HAp, grafting densities of above 2 µg/cm^2^ were required [78].

Phosphonate functional groups can also be introduced by the chemical treatment of polymers. Mahjoubi et al. modified poly(D,L-lactic acid) (PDLLA) surface with phosphonate groups via diazonium chemistry [81]. The immersion in c-SBF for 2 and 4 weeks resulted in a HAp-like coating (Ca/P ratio of 1.7) that contained crystals with globular morphology, covering the entire PDLLA surface [81]. By calculating the ratio of v1(PO_4_^3−^):v(C–COO) from the Raman spectra, it was demonstrated that the coating thickness increased with incubation time in the SBF. The Fourier transfer infrared (FTIR) assessment revealed the presence of v3(CO_3_^2−^) peaks between 1400 and 1600 cm^−1^, and v1 and v3(PO_4_^3−^) peaks between 900 and 1000 cm^−1^, which were the IR signature of stoichiometric HAp. Both chondrogenic cell line, ATDC5, and osteoblastic cell line, MC3T3-E1, showed good biocompatibility with the HAp-coated PDLLA and higher mineral deposition rate than when cultured on the non-coated substrate. Another example of phosphorylation technique was performed by Sailaja et al. by incubating PVA films in phosphoric acid and urea [82]. After 10 days of incubation in c-SBF, layers of HAp (Ca/P ratio of 1.67) could be found on the phosphorylated PVA surface [82]. However, the X-ray diffraction (XRD) pattern of the coating suggested a poor HAp crystallinity. Nevertheless, human osteosarcoma cells were shown to attach well on the coated-PVA films and have a higher mineralization rate (higher von Kossa staining intensity) than when cultured on untreated PVA films.

#### 4.1.2. Biomineralization on Carboxylated and Hydroxylated Surfaces

**Figure 2:**
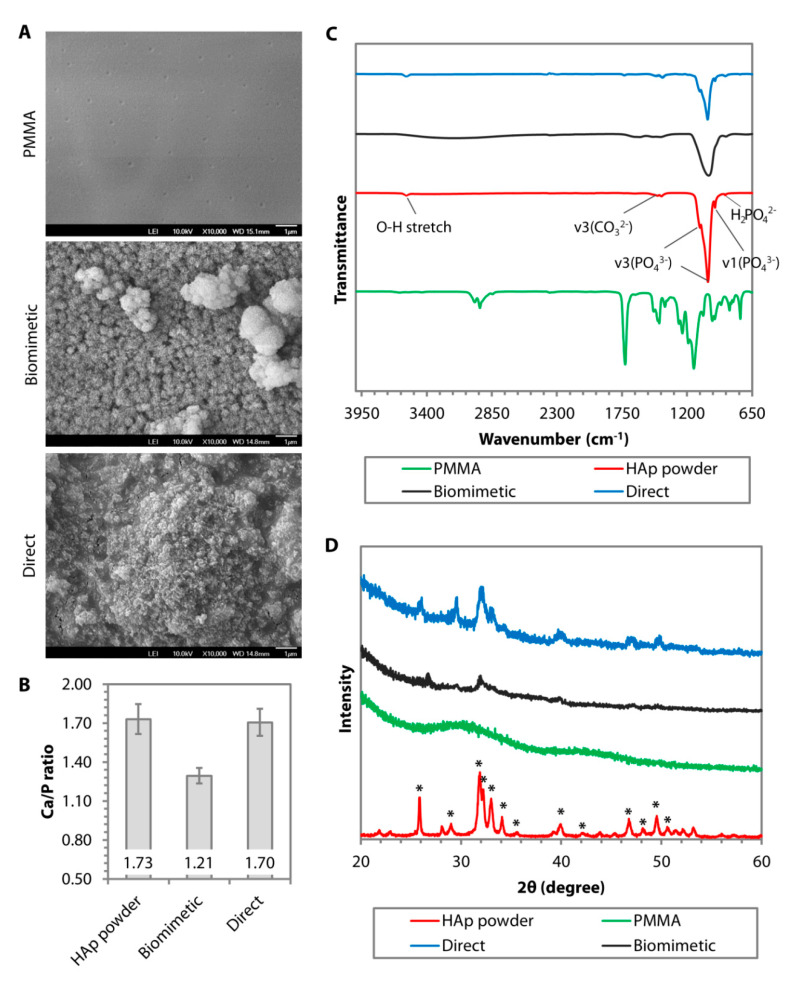
Comparison of surface morphology, chemical composition, phase, and crystallinity of hydroxyapatite (HAp) coating deposited on poly(methyl methacrylate) (PMMA) via biomimetic deposition or direct immobilization approach. (**A**) Scanning electron microscopy (SEM) images of PMMA surface before and after HAp coating via biomimetic deposition or direct immobilization technique. (**B**) Ca/P ratio generated from the energy dispersive X-ray (EDX) of calcined HAp (stoichiometric HAp) and the resulting CaP minerals deposited on the PMMA. (**C**) Fourier transform infrared (FTIR) patterns of uncoated PMMA (in green) and stoichiometric HAp (in red) showed a distinct IR band difference between the groups. A peak of v3(PO_4_^3−^) was found in after either biomimetic (in black) or direct (in blue) deposition. However, the other v3(PO_4_^3−^) and v1(PO_4_^3−^), which were typical of stoichiometric HAp, could only be seen in the direct deposition group. (**D**) Graze incidence-X-ray diffraction (GI-XRD) pattern revealed that the calcined HAp powder exhibited the prominent characteristic peaks of pure HAp according to JCPDS no. 00-009-0432. Most of the peaks also appeared in the direct immobilization group. In contrast, the biomimetic group exhibited an XRD pattern of amorphous octacalcium phosphate (OCP).

The other effective surface functionalizations to induce biomineralization are carboxylation and hydroxylation. Tretinnikov et al. grafted poly(acrylic acid) (PAAc) on HDPE before subjecting the HDPE to apatite deposition in c-SBF [78]. The authors found that although the deposition rate was slower than on MOEP-grafted HDPE, the Ca/P ratio of the apatite was higher (ranged between 2.2 and 2.6), suggesting an excess binding of Ca^2+^ [78]. Cui et al. showed that biomineralization in 2× SBF was more favorable on electrospun PDLLA substrate functionalized with –COOH groups or the combination of –OH and –COOH groups with a molar ratio of 3/7 or –NH_2_, –OH and –COOH groups with a molar ratio of 2/3/5 [83]. XRD identified the coating as HAp as early as 7 days after incubation in the SBF and the crystallinity improved with longer immersion time [83]. Another example of –COOH functionalization via chemical treatment was found in studies by Wang et al. and our group [16,22]. Biomineralization was observed on PMMA, pretreated with a combination of polydopamine and 11-mercaptoundecanoic acid (11-MUA), after a 14-day incubation in 1.5× SBF (Figure 2A). In contrast to the smooth and almost featureless surface of untreated PMMA, we noted the presence of crystals with globular morphology, forming a calcium-deficient apatite layer (Ca/P ratio of 1.21 ± 0.03) on the carboxylated PMMA surface (Figure 2A,B). FTIR revealed a distinct peak at 1029 cm^−1^, suggesting the presence of v3(PO_4_^3−^) (Figure 2C). However, two other peaks at 1147 cm^−1^ (v3(PO_4_^3−^)) and 960 cm^−1^ (v1(PO_4_^3−^)) that were characteristics of stoichiometric HAp (Figure 2C), could hardly be detected. A previous study has shown that low resolution of v1 and v3(PO_4_^3−^) IR bands were typically an indication of poor crystallinity of an apatite coating [84]. Using grazing incidence-X-ray diffraction (GI-XRD) at 1° grazing angle, we confirmed that the coating was indeed rather amorphous. There was a broad area under the curve, especially in the region beneath the most prominent peak at 2θ of 31.9° (Figure 2D). Another prominent peak was detected at 2θ of 26.1° **(**Figure 2D). According to JCPDS no. 00-026-1056, these two peaks suggested that the deposited CaP minerals were octacalcium phosphate (OCP), which was consistent with our EDX result (Figure 2B). In spite of that, the corneal stromal fibroblasts appeared to have higher attachment efficiency, proliferation, and survival rate on the coated PMMA than on the untreated surface [22].

Recently, Permyakova and colleagues functionalized electrospun PCL nanofibers with –COOH by using atmospheric pressure plasma copolymerization of CO_2_ and C_2_H_4_, followed by biomineralization of the PCL in c-SBF for 21 days [85]. The authors showed a stable and linear increase in Ca concentrations over 21 days and complete coverage of the PCL nanofibers by 14 days. In contrast, the pristine PCL showed a fluctuation in the Ca concentrations and poor biomineralization over the 21-day incubation in c-SBF. There was no XRD analysis performed in the study to resolve the crystallinity of the CaP coating. The CaP coating significantly improved the adhesion and proliferation of IAR-2 epithelial cells, but not the MC3T3-E1 osteoblasts.

Hydroxylation, although not as effective as carboxylation, has also been carried out to induce biomineralization on low T_m_ polymers. Two of the most frequently found hydroxylation techniques in the literature are oxygen plasma treatment and chemical treatment with sodium hydroxide (NaOH) [16,22,83,86,87,88]. Qu et al. and our group showed that the biomineralization outcomes were similar between oxygen plasma-treated PCL and PMMA in 1.5× SBF [22,88]. Both found that the biomineralization process resulted in calcium-deficient apatite minerals with a rather poor level of crystallinity. Qu et al. further showed that OCT-1 osteoblast-like cells had a significantly better attachment efficiency and a marginally better proliferation on the coated PLGA than on the non-coated PLGA [88]. There was no difference in the alkaline phosphatase (ALP) activity between the non-coated and coated PLGA. In a separate study, Oyane et al. found that NaOH of 1M to perform surface hydrolysis on PCL was required to induce an effective biomineralization activity in c-SBF [87]. The rate of CaP deposition was commensurate with the increase of the NaOH concentration used to base hydrolyze the polymer [75]. However, GI-XRD showed that the coating had low crystallinity regardless of the NaOH concentration.

Murphy et al. attempted to perform biomineralization of poly(lactide-co-glycolide) (PLG), which surface had been pre-activated with 0.5M NaOH, in m-SBF (142.0 mM Na^+^, 5.0 mM K^+^, 1.5 mM Mg^2+^, 2.5 mM Ca^2+^, 103.0 mM Cl, 10.0 mM HCO^3−^, 1.0 mM HPO_4_^2−^, and 0.5 mM SO_4_^2−^) for 7 days [86]. The coating resulted in calcium-deficient HAp with a Ca/P ratio of 1.55. The study did not present any in-depth surface chemistry analysis with XRD or FTIR. Human mesenchymal stem cells, seeded on the biomineralized PLG, had a higher proliferation rate, but lower ALP activity and osteocalcin production than when seeded on the pristine PLG.

#### 4.1.3. Biomineralization on Peptide-bound Surface

Material-binding peptides have recently been used as non-covalent bound linkers on polymers [89,90]. The peptides can add certain functions to the polymers by permitting a further conjugation with functional molecules, such as biotin, bioactive peptides, or enzymes [89,90]. Due to the relatively recent discovery, only one example of the relevant application could be found in the literature [91]. The CaP mineralization was performed on a polymer with T_m_ above 200 °C. However, we can assume that the technique could also be applied to a lower T_m_ polymer, considering that peptides with specific binding motifs to PMMA and polycarbonate (PC) have been brought to light [92]. In the study by Iijima et al. [91], they showed that surface functionalization of polyetherimide (PEI) with peptide conjugates with sequences of PEI-binding peptide (TGADLNT-EG_2_-DDD) induced biomineralization in 1.5× SBF. EG_2_ or diethylene glycol unit, originated from [2-[2-(Fmoc-amino)ethoxy]ethoxy]acetic acid, is a bifunctional crosslinker that was used to link the CaP mineralization-promoting sequence (DDD) to PEI-binding peptide. However, on FTIR, only one v3(PO_4_^3−^) peak and no v1(PO_4_^3−^) peak between 1000 and 1200 cm^−1^ were detected in the apatite-like coating. The Ca/P ratio was not reported.

Utilizing the versatility of polydopamine as an adhesive molecule [93], Ghorbani et al. functionalized freeze-casted PCL scaffolds via a 24-h dip-coating in the polydopamine solution [94]. Biomineralization of the scaffolds was carried out in the c-SBF solution for 28 days under constant rotation of 30 rpm. On XRD, the existence of peaks at 2θ angle of 22.9°, 25.6°, 31.5°, 45.4°, and 56.4° confirmed the formation of HAp coating (JCPDS no. 00-09-0432), although the Ca/P ratio was only 1.46. The HAp coating resulted in significantly better adhesion, viability, and proliferation of L-929 fibroblasts, as well as better osteoinduction as evidenced by the higher level of alkaline phosphatase secretion from MG-63 cells. Substantiating the beforementioned study, Zhang et al. demonstrated that the biomineralized polydopamine-activated PCL nanofibers were also biocompatible to M3T3-E1 cells and induced better osteoconduction compared to pristine PCL [95]. In a mechanistic study, Ryu et al. showed that the terminal –OH groups of the polydopamine were responsible in initiating the biomineralization activity on various biomedical polymers, e.g., PMMA, polystyrene (PS), and polydimethylsiloxane (PDMS) [96]. XRD pattern suggested that the deposited minerals were HAp rather than OCP (JCPDS no. 00-026-1056). An innovative diffusion-controlled oxygen supply technique was employed by Perikamana et al. to functionalize PLLA nanofibers with polydopamine in a gradient manner [97]. The authors were able to demonstrate on XRD that the regions with higher concentrations of polydopamine tended to have a higher HAp mineralization rate (JCPDS no. 00-09-0432). It is worth noting the activation of dopamine results in a brownish film on substrates and therefore, limits its application for corneal tissue engineering that typically requires transparent substrates.

### 4.2. Direct Nanoparticle Immobilization Approach

Inconsistencies in the biomineralization process in SBF, and the phase and crystallinity of the resulting CaP coating, are likely attributed to the variations in the surface functionalization techniques. The coating outcomes are also sensitive to changes in pH and temperature of the SBF solution, which undoubtedly would occur during the relatively long period of incubation time and SBF storage [98,99]. These limitations prompted us to find an alternative approach to deposit CaP nanoparticles on the surface of polymers. The approach was formulated to circumvent the surface functionalization step and significantly shorten the time to deposit the coating, as well as to utilize calcined or annealed HAp nanoparticles (to produce a coating that mimics the stoichiometric HAp). This contrasts with the conventional dip-coating method that is briefly mentioned in Section 3.4, whereby the substrate is dipped in supersaturated CaP solutions. As such, the CaP coating is typically amorphous and not in the stoichiometric HAp form. Annealing the CaP deposits at a temperature of 1000 °C is necessary to produce a crystalline coating [100]. The direct immobilization technique via dip-coating was inspired by the solvent casting technique, performed by Wang and colleagues [101]. In their study, calcium-deficient hydroxyapatite (CDHA) nanocrystals, dispersed in dimethylformamide (DMF) and PLA, were deposited on a metallic substrate by solvent casting method. The CDHA was observed to be homogeneously distributed in 0.1-thick PLA films following solvent evaporation and had similar morphology and composition to natural bone mineral.

**Figure 3:**
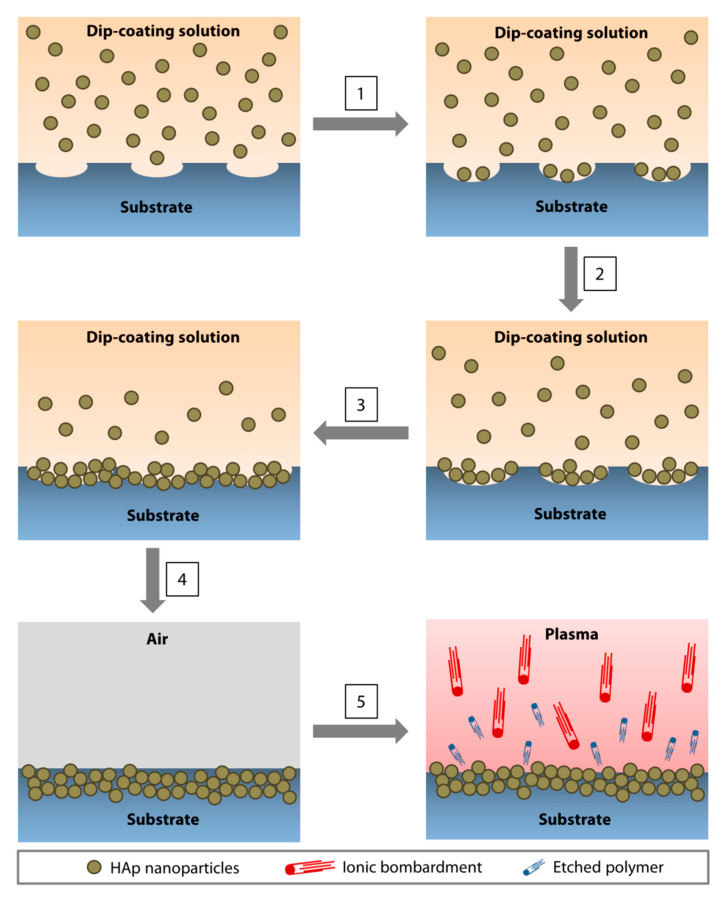
Direct immobilization of HAp nanoparticles via dip-coating method. (**1**) As the polymeric surface was softened by the organic solvent, pores started to form, followed by the deposition of the nanoparticles in the pores. (**2**,**3**) The pores enlarged as the dip-coating progressed and an increasing amount of nanoparticles accumulated in the cavities. (**4**) At the conclusion of the dip-coating, the substrate was air-dried. During the drying process, the ‘liquified’ surface began to resolidify, immobilizing the nanoparticles near the surface of the substrate. (**5**) Oxygen plasma treatment resulted in ion bombardment of the surface, removing contaminants, and etching away the polymer that masked the nanoparticles during the drying step. An increasing amount of HAp nanoparticles emerged as the residual polymer was etched.

The nanoparticle immobilization was achieved via dip-coating of a polymeric substrate in an organic solution (to soften or ‘liquify’ the surface of the substrate to allow seeding of nanoparticles) containing HAp nanoparticles and a low amount of polymer (to increase the viscosity of the organic solution to slow the nanoparticle agglomeration and produce a more uniform coating) (Figure 3) [102]. After drying, the substrate was subjected to oxygen plasma etching for 5 min to remove surface contaminants and residual polymer that may mask the superficial layer of the coating (Figure 3). In our application, a PMMA substrate was dip-coated in chloroform containing 5% (*w/v*) of PMMA and 20% (*w/v*) of 60-nm HAp nanoparticles. We found that a single 1-min dip was optimal to coat a flat surface of PMMA sheets [102]. Coating a curved and smaller surface area of PMMA rods required multiple 5-s dips (up to 12 times) [26]. The reason behind the difference is currently unknown. The elucidation of the precise mechanism will require molecular dynamic simulations.

**Figure 4:**
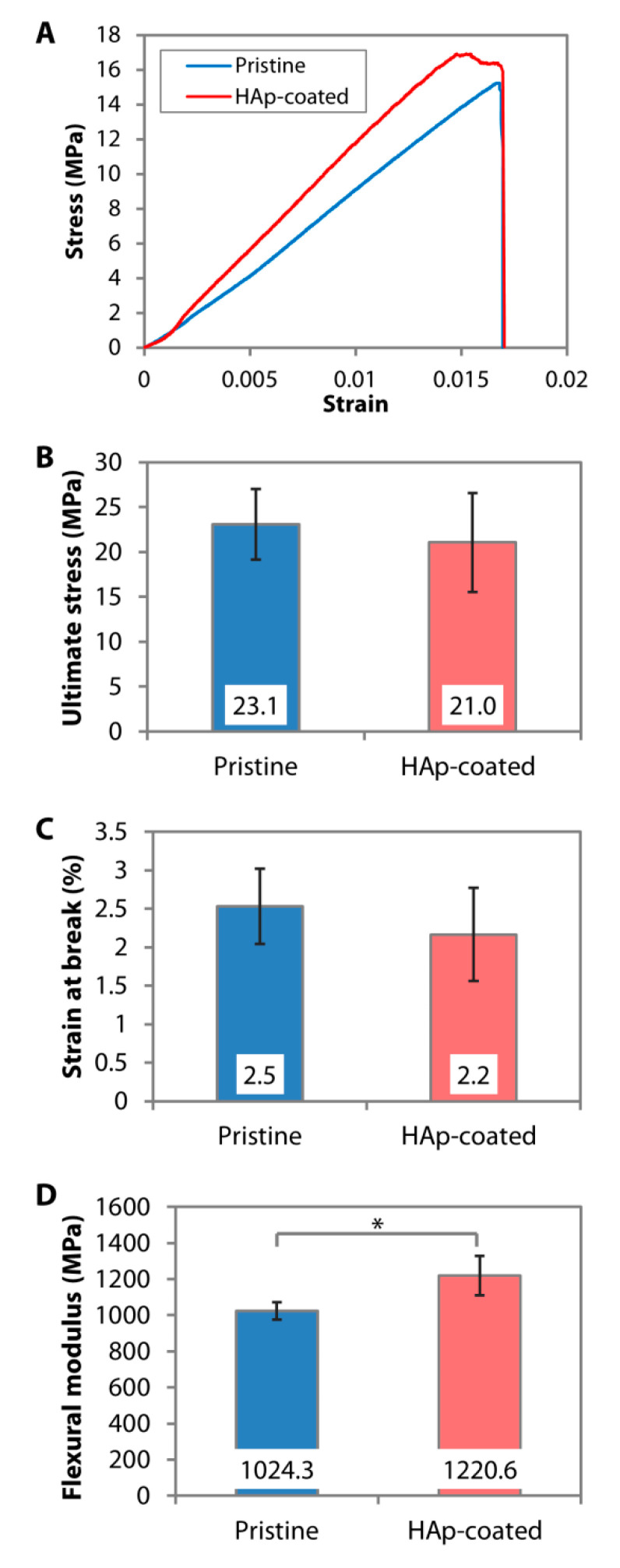
Mechanical properties of pristine and dip-coated PMMA. HAp nanoparticles were immobilized on the PMMA sheets via dip-coating. (**A**) Representative stress-strain curves generated from 3-point bending tests of the PMMA sheets. (**B**–**D**) Ultimate flexural stress, strain at break, and flexural modulus of pristine and dip-coated PMMA sheets. * *p* < 0.05.

The dip-coating resulted in a relatively rough surface interspersed with HAp nanoparticles (Figure 2A). The resulting coating had a similar Ca/P ratio (Figure 2B) and IR pattern (Figure 2C) to the calcined HAp. Peaks attributed to O−H stretch, v3(CO_3_^2−^), v1(PO_4_^3−^), v3(PO_4_^3−^), and v(HPO_4_^2−^) could be found on both the calcined HAp and the dip-coated PMMA surface. Although it was difficult to determine the crystallinity level of the coating from the GI-XRD (due to the signal interference from the PMMA that filled the gaps between the nanoparticles), the major peaks of calcined HAp or stoichiometric HAp (JCPDS no. 00-009-0432) were noticeable on the dip-coated PMMA surface, e.g., peaks at 2θ of 25.8°, 29.0°, 31.9°, 33.0°, 34.1°, 39.9°, 46.7°, and 49.5° (Figure 2D). The area under 2θ = 31.9° peak did not appear as broad as that seen in the XRD patterns of amorphous PMMA and SBF-mediated CaP deposition, suggesting that the crystallinity was mostly unaffected by the dip-coating and oxygen plasma treatment. By carrying out a 3-point bending test on the PMMA sheets, we found that the ultimate stress (*p* = 0.481) and strain at break (*p* = 0.279) were similar to the pristine PMMA (Figure 4A–C). Due to the presence of 30-to-50-µm-thick HAp layers laminating the PMMA surface [102], the coated PMMA was significantly stiffer than the pristine PMMA (*p* = 0.005) (Figure 4D).

## 5. Conclusions and Future Perspectives

Over the last three decades, there have been significant advances made in improving the efficiency of CaP deposition on low T_m_ polymers via the biomimetic route. The advantages of the biomimetic approach include the simplicity of the method, the obviation of specialized equipment, and the possibility to coat porous substrates or substrates with complex dimensions. However, the lack of SBF use in clinical practice indicates the need for further research and improvements to overcome these translational challenges. The dearth of publications in this specific area of research for the past five years is another proof of the stagnation of the translational efforts. This could be due to several factors. The coating outcomes have been inconsistent between studies in terms of the crystallinity level, CaP phase, and mineral purity. Several studies found that the resulting minerals were non-stoichiometric HAp and amorphous [22,78,79,82,88]. CaP minerals with such properties possess higher solubility than HAp and, therefore, are less suitable for potential long-term clinical uses [103,104]. Since SBF contains many other ions, chemically pure HAp cannot be precipitated from the incubation in the solution. Ion-substituted CDHA is the predominant CaP form that is precipitated instead. The inconsistencies of the properties of the coating in many reports are an issue that could be largely attributed to the effect of the different surface functional groups that have to be introduced to induce an efficient apatite nucleation process, as well as the fluctuations in pH and temperature of the SBF (in the preparation, storage and/or during the coating process) [98,99]. In SBF, the CaP deposition takes a relatively long time (usually days to weeks) to build up and cover the entire surface of polymers. Because the CaP coating is anchored by ionic bonds to the substrate, delamination can easily occur, especially on devices that frequently experience tangential or horizontal forces [9]. Upscaling a process that is both inconsistent and takes a long time is not feasible from a practical point of view.

The direct immobilization technique via dip-coating was developed to circumvent some of the aforementioned limitations of the biomimetic CaP deposition. An advantage of the technique is that the fidelity of the phase, crystallinity, and purity of the calcined HAp is maintained in the coating. Hence, the expected bioactivity of the HAp or other nanoparticles is not lost or reduced when immobilized on the polymers [102,105]. The superior resistance to biodegradation of HAp compared to other CaP phases may allow longer-term stability of the polymeric implants in vivo. Besides this, the dip-coating and subsequent plasma etching can be carried out in 1 day. Since most low T_m_ polymers are soluble in organic solvents, the technique is potentially applicable to those polymers. It may also apply to porous polymers provided the pore sizes are not significantly smaller than the nanoparticles. However, a series of optimization of the concentration of nanoparticles and polymer, the size of nanoparticles, the type of solvent, and the length of dip-coating may have to be performed due to the variations in nanoparticle interaction dynamics and the dissolution rate of different polymers. 

The therapeutic effects of the coating deposited via the biomimetic route can be diversified by co-precipitating growth factors/proteins or DNA in the SBF [106,107]. The therapeutic effect of the coating deposited by the direct immobilization method is dependent on the bioactivity produced by the nanoparticles used. For example, a mixture of immobilized silver and HAp nanoparticles offers anti-bacterial and improved biocompatibility effects to the polymer [105]. Due to the likelihood of proteins or DNA degradation in the organic solvent, any functional addition with proteins or DNA cannot be carried out simultaneously with the dip-coating. The advantages and disadvantages of direct immobilization and biomimetic HAp deposition are summarized in Table 2.

The American Society for Testing and Materials (ASTM) standards specification F1185-03 states that surgical implants require at least 95% of HAp content, established by XRD analysis, while the concentration of trace elements have to be limited to 3 ppm of arsenic, 5 ppm of cadmium, 30 ppm of lead, and 5 ppm of mercury [108]. The Ca/P ratio of HAp used for surgical implants must be between 1.65 and 1.82 [108]. Additionally, the International Organization of Standards (ISO) stated in ISO 13779 that it requires the HAp coating on implants to exhibit a crystallinity of at least 45% with the maximum allowable limit of all heavy metals at 50 ppm [109]. Although the above standards are currently applied to control the quality and safety of thermal sprayed coating, they can serve as a guideline for the application of non-thermal HAp coating on polymeric products intended for tissue engineering in the future. This offers a translational advantage to the direct immobilization method as the coating procedure does not cause any alteration to the intrinsic properties of the HAp nanoparticles, which have been fine-tuned and synthesized conforming to the ASTM and ISO standards.

In summary, the direct immobilization technique offers advantages of a shorter coating time, obviation of the need for surface functionalization of substrates, and consistency of the crystallinity and mineral phase of stoichiometric HAp in the coating. We have previously optimized the coating method in PMMA. However, due to the novelty of the technique, optimization will be necessary to create a uniform coating on other polymers. It also remains to be explored whether it is possible to apply the technique to polymers with higher T_m_, e.g., polyether ether ketone (PEEK; T_m_ = 343 °C) or polytetrafluoroethylene (PTFE; T_m_ = 327 °C) [110]. The possibilities to coat porous polymers and polymers with complex dimensions will also need to be researched in the future to expand the applications. In contrast, SBF-mediated CaP deposition has been extensively studied in the literature and can be readily applied to any polymer, although the outcomes can be somewhat unpredictable. The route to the clinical translation of either coating approach is still long and arduous. Currently, in vivo animal experiments are needed to support the in vitro work and determine the safety and performance of both coatings.

## Figures and Tables

**Table 1:** Composition of simulated body fluid (SBF) and its variants.

Solution	Ionic Concentration (mM)								Buffer, pH	Reference
	Na^+^	K^+^	Mg^2+^	Ca^2+^	Cl^−^	HCO^3−^	HPO_4_^2−^	SO_4_^2−^		
Blood plasma	142.0	5.0	1.5	2.5	103.0	27.0	1.0	0.5	-	[66]
Original SBF	142.0	5.0	1.5	2.5	148.8	4.2	1.0	0	* Tris, 7.25–7.4	[65]
c-SBF	142.0	5.0	1.5	2.5	147.8	4.2	1.0	0.5	Tris, 7.25–7.4	[66]
r-SBF	142.0	5.0	1.5	2.5	103.0	27.0	1.0	0.5	** HEPES, 7.4	[70]
np-SBF	142.0	5.0	1.5	2.5	103.0	4.2	1.0	0.5	HEPES, 7.4	[68]
t-SBF	142.0	5.0	1.5	2.5	125.0	27.0	1.0	0.5	*** dH_2_O	[67]
i-SBF	142.0	5.0	1.0	1.6	103.0	27.0	1.0	0.5	HEPES, 7.4	[69]
m-SBF	142.0	5.0	1.5	2.5	103.0	10.0	1.0	0.5	HEPES, 7.4	[69]
1.5× SBF	213.0	7.5	2.3	3.8	223.0	6.3	1.5	0.75	Tris, 7.25	[72]
5× SBF	726.0	25.0	7.5	12.5	760.0	21.0	5.0	2.5	Tris, 7.4	[71]
10× SBF	1020.0	5.0	5.0	25.0	1035.0	10.0	10.0	-	dH_2_O	[73]

* Tris = 2-amino-2-hydroxymethyl-propane-1,3-diol; ** HEPES = 2-[4-(2-hydroxyethyl)piperazin-1-yl]ethanesulfonic acid; *** dH_2_O = deionized water.

**Table 2:** Advantages and disadvantages of biomimetic deposition and direct immobilization of HAp techniques.

Biomimetic	Direct Immobilization
Does not need specialized equipment.	Requires an automated dip-coater and a plasma cleaner.
Lower cost.	Higher cost of materials, especially the nanoparticles.
Requires surface functionalization before biomineralization process to enhance the nucleation efficiency.	Does not require surface functionalization before dip-coating.
Relatively long biomineralization time (can take days to weeks).	Short coating time (1 day).
Inconsistent coating outcomes in terms of CaP phase, crystallinity, and purity.	Fidelity of HAp phase, crystallinity, and purity is maintained in the coating.
Relatively low coating adhesion to substrate (mostly ionic bonds).	Better coating adhesion due to physical immobilization of HAp into the substrate.
Due to the aqueous nature of SBF, the coating can be applied to polymers with pores and complex dimensions.	May be applicable to polymers with pores and complex dimensions (extensive optimization has to be carried out).
Can be co-precipitated with growth factors/proteins or DNA to add additional therapeutic effects.	Unlikely to avoid degradation of proteins or DNA that are tethered on the nanoparticles in the organic solvent; hence, the therapeutic function is limited to that provided by the nanoparticles.
